# The silent crisis: effect of malnutrition and dehydration on children in Gaza during the war

**DOI:** 10.3389/fnut.2024.1395903

**Published:** 2024-04-22

**Authors:** Samer Abuzerr, Ayoub Al-Jawaldeh, Yara Ashour, Kate Zinszer, Abdel Hamid El Bilbeisi

**Affiliations:** ^1^Department of Medical Sciences, University College of Science and Technology-Khan Younis, Gaza, Palestine; ^2^Regional Office for the Eastern Mediterranean (EMRO), World Health Organization (WHO), Cairo, Egypt; ^3^Department of General Surgery, Al-Quds University, Gaza, Palestine; ^4^Department of Social and Preventive Medicine, School of Public Health, University of Montreal, Montréal, QC, Canada; ^5^Department of Nutrition, School of Medicine and Health Sciences, University of Palestine, Gaza, Palestine

**Keywords:** silent crisis, malnutrition, dehydration, children, war, Gaza Strip

## Introduction

In the midst of conflict, the plight of vulnerable populations often remains hidden beneath the rubble and chaos. However, the recent escalation of violence in the Gaza Strip has thrust the dire humanitarian situation into the spotlight, particularly highlighting the devastating impact on children. Prior to the current conflict, more than 75% of the population in Gaza relied on humanitarian assistance for basic necessities, a stark indication of the precariousness of their situation (1).

## Discussion

The ongoing conflict has exacerbated existing vulnerabilities, with food and safe water becoming increasingly scarce commodities. The intensification of airstrikes, frequent border closures, and restrictions on aid delivery have further compounded the crisis, leaving hundreds of thousands of people in Gaza facing extreme hunger and malnutrition. As a result, diseases are spreading rampantly, compromising the nutrition and immunity of women and children, and leading to a surge of acute malnutrition across the region.

Recent reports have revealed alarming statistics, painting a grim picture of the situation in Gaza. Nutrition screenings conducted in the Northern Gaza Strip found that 15.6% of children under 2 years of age are acutely malnourished, surpassing the critical threshold set by the World Health Organization. The situation is particularly dire in the Northern Gaza Strip, where aid has been almost completely cut off for weeks, exacerbating the suffering of the most vulnerable populations (2).

Tragically, the consequences of malnutrition extend beyond individual lives, with profound societal implications. The Integrated Food Security Phase Classification has warned of a very high risk of famine in Gaza, with at least 1 in 4 households facing catastrophic levels of acute food insecurity. Pregnant women and breastfeeding mothers are particularly vulnerable, facing increased risks of malnutrition and death due to limited access to food, water, and medical supplies (3).

The impact of malnutrition and dehydration on children in Gaza is devastating, with reports indicating a dramatic increase in child deaths due to acute malnutrition (4). The situation is exacerbated by the lack of access to safe water, with over 90% of children affected by one or more infectious diseases and 70% experiencing diarrhea in the past 2 weeks (4).

Urgent action is needed to address the humanitarian crisis unfolding in Gaza. Immediate humanitarian ceasefire is essential to allow the delivery of life-saving aid and the restoration of essential services across the region. This includes the provision of infant milk, food and nutrient supplements, ready-to-use therapeutic foods, water, medical supplies, and fuel. Additionally, protecting hospitals and health workers from attack is imperative to ensure critical treatment and care for the most vulnerable (5).

## Conclusion

To address food insecurity and famine in the Gaza Strip effectively, a comprehensive approach involving diverse stakeholders, such as UNRWA, WFP, FAO, WHO, UNICEF, and others, is imperative. This necessitates concerted efforts to establish sustainable and equitable systems for the delivery of food, water, and health services, while simultaneously enhancing the capacity of local authorities to address malnutrition among vulnerable populations.

Continued support for affected communities is recommended to aid in their recovery, foster social cohesion, and fortify resilience against future crises. Mechanisms for financial protection should be devised to safeguard individuals in emergency contexts, with concerted efforts aimed at mobilizing and coordinating resources to ensure the availability of flexible funding to address urgent priorities. Moreover, the adaptation and review of contingency funding mechanisms are advised to prioritize essential food, nutrition, and medicine requirements during ongoing emergencies.

Prioritizing the integration of food security and nutrition interventions into national emergency response plans within the food and health sectors is paramount for ensuring a seamless continuum of food supply and essential nutrition services during crises. Sustained access to vital food aid and medicines, including specialized kits and technologies for managing acute malnutrition at health facilities, must be guaranteed to mitigate the adverse impacts of emergencies effectively.

## Author contributions

SA: Supervision, Validation, Writing—original draft, Writing—review & editing. AA-J: Writing—original draft, Writing—review & editing. YA: Writing—original draft, Writing—review & editing. KZ: Data curation, Methodology, Validation, Writing—review & editing. AE: Writing—original draft, Writing—review & editing.
